# Novel and *de novo* mutations in pediatric refractory epilepsy

**DOI:** 10.1186/s13041-018-0392-5

**Published:** 2018-09-05

**Authors:** Jing Liu, Lili Tong, Shuangshuang Song, Yue Niu, Jun Li, Xiu Wu, Jie Zhang, Celement C. Zai, Fang Luo, Jian Wu, Haiyin Li, Albert H. C. Wong, Ruopeng Sun, Fang Liu, Baomin Li

**Affiliations:** 1 Department of Pediatrics, Qilu Hospital of Shandong University, Jinan, Shandong People’s Republic of China; 2 Shandong University, Jinan, Shandong People’s Republic of China; 3 Qilu Children’s hospital of Shandong University, Jinan, Shandong People’s Republic of China; 4 MyGenostics Inc., Beijing, People’s Republic of China; 5 Campbell Family Mental Health Research Institute, Centre for Addiction and Mental Health, University of Toronto, Toronto, ON Canada

**Keywords:** Refractory epilepsy, Next-generation sequencing, ACMG scoring

## Abstract

Pediatric refractory epilepsy is a broad phenotypic spectrum with great genetic heterogeneity. Next-generation sequencing (NGS) combined with Sanger sequencing could help to understand the genetic diversity and underlying disease mechanisms in pediatric epilepsy. Here, we report sequencing results from a cohort of 172 refractory epilepsy patients aged 0–14 years. The pathogenicity of identified variants was evaluated in accordance with the American College of Medical Genetics and Genomics (ACMG) criteria. We identified 43 pathogenic or likely pathogenic variants in 40 patients (23.3%). Among these variants, 74.4% mutations (32/43) were *de novo* and 60.5% mutations (26/43) were novel. Patients with onset age of seizures ≤12 months had higher yields of deleterious variants compared to those with onset age of seizures > 12 months (*P* = 0.006). Variants in ion channel genes accounted for the greatest functional gene category (55.8%), with *SCN1A* coming first (16/43). 81.25% (13/16) of *SCN1A* mutations were *de novo* and 68.8% (11/16) were novel in Dravet syndrome. Pathogenic or likely pathogenic variants were found in the *KCNQ2*, *STXBP1*, *SCN2A* genes in Ohtahara syndrome. Novel deleterious variants were also found in West syndrome, Doose syndrome and glucose transporter type 1 deficiency syndrome patients. One *de novo* *MECP2* mutation were found in a Rett syndrome patient. *TSC1/TSC2* variants were found in 60% patients with tuberous sclerosis complex patients. Other novel mutations detected in unclassified epilepsy patients involve the *SCN8A, CACNA1A, GABRB3, GABRA1, IQSEC2, TSC1, VRK2, ATP1A2, PCDH19, SLC9A6* and *CHD2* genes. Our study provides novel insights into the genetic origins of pediatric epilepsy and represents a starting-point for further investigations into the molecular pathophysiology of pediatric epilepsy that could eventually lead to better treatments.

**Electronic supplementary material:**

The online version of this article (10.1186/s13041-018-0392-5) contains supplementary material, which is available to authorized users.

## Introduction

Epilepsy is a complex group of chronic brain disorders that are characterized by recurrent spontaneous seizures, and these can often begin in childhood. Repeated and refractory seizures can cause long-term cognitive impairment, decreased social participation and significantly lower quality of life [1, 2]. Epilepsy is one of the most common neurological disorders with 50 to 100 million affected worldwide, and 2 to 4 million new cases diagnosed each year [3].

Epilepsy is a heterogeneous disease with diverse clinical manifestations and causes, including altered ion channel expression, neurotransmitter signaling, synaptic structure, gliosis, and inflammation [1]. Estimates of heritability from twin studies range from 25% to 70% [4, 5]. Although the range of heritability estimates is quite large, disparate studies using varied methods and studying divergent populations are all consistent in concluding that there is a substantial inherited component to epilepsy [6]. Because of this, we sought to investigate the genome in a heterogeneous set of patients with epilepsy and their parents, with the hope that we would identify novel mutations and confirm existing reports of genetic associations with epilepsy. This type of genetic information can provide an entry point into the biology of epilepsy that could eventually lead to new molecular treatment targets.

With the rapid progress of next-generation sequencing (NGS) techniques, our knowledge of the genetic etiology in many brain disorders such as epilepsy, autism and intellectual disability has expanded greatly [7, 8]. NGS is now capable of efficient and accurate sequencing of entire genomes with small amounts of tissue at ever decreasing costs and has required new approaches to analysing the very large amount of data obtained. For this study, our priority was to separate common and benign genetic variants from those that are likely to be related to the cause of epilepsy, and we chose to apply the American College of Medical Genetics and Genomics (ACMG) guidelines [9–11]. The ACMG guidelines classify variants into pathogenic, likely pathogenic, uncertain significance, likely benign, and benign categories based on genetic information that includes population, functional, computational and segregation data. In this study, we investigated 153 epilepsy candidate genes in a cohort of 172 refractory epilepsy pediatric patients. We aimed to provide genetic diagnoses of this patient cohort and explore the genetic etiology of pediatric refractory epilepsy.

## Method

### Participants

We retrospectively collected and analyzed 172 cases of pediatric refractory epilepsy patients between the ages of 1 day to 14 years old in the Department of Pediatrics of Qilu Hospital, China. The program adhered to guidelines of patients’ consent for participation and research was supported by the Ethics Committee of Qilu hospital, Shandong University (No. 2016(027)).

All patients were examined and diagnosed at the Pediatric Department in Qilu Hospital using a combination of patients’ illness history, previous history, family history, physical examinations, developmental evaluation, hematological examination, ambulatory or video electroencephalography (AEEG/VEEG) monitoring, magnetic resonance imaging (MRI) or computed tomography (CT), and genetic sequencing. Developmental evaluation included gross motor, fine motor, language, and personal-social skills. The above information was reviewed by two qualified pediatric epileptologists. Seizure types and epilepsy syndromes were diagnosed and classified according to the guidelines of International League Against Epilepsy (2014, 2017) [12, 13].

### Next-generation sequencing

#### Targeted gene capture and sequencing

Blood samples of the patients and their biological parents were collected to test if the mutations were *de novo* or inherited. Genomic DNA was extracted from peripheral blood using the QIAamp DNA Mini Kit (Qiagen, China).

One hundred fifty-three genes (Table 1) associated with epilepsy were selected by a gene capture strategy, using the GenCap custom enrichment kit (MyGenostics, China) following the manufacturer’s protocol. The biotinylated capture probes were designed to tile all of the exons without repeated regions. The captured DNAs were eluted, amplified and then their polymerase chain reaction (PCR) products were purified with SPRI beads (Beckman, USA). The enriched libraries were sequenced for paired-end reads of 150 bp by Illumina HiSeq X Ten.

**Table 1:** One hundred fifty-three epilepsy genes tested in this study by NGS

*ADSL*	*CHD2*	*DHFR*	*GLB1*	*MAGI2*	*PNPO*	*SLC9A6*
*ALDH7A1*	*CHRNA2*	*DIAPH3*	*GLRA1*	*MAPK10*	*POLG*	*SPTAN1*
*ALG13*	*CHRNA4*	*DNAJC6*	*GPR56*	*MBD5*	*PPT1*	*SRPX2*
*ARG1*	*CHRNA7*	*DNM1*	*GPR98*	*MDGA2*	*PROC*	*ST3GAL2*
*ARHGEF15*	*CHRNB2*	*DOCK7*	*GRIN1*	*ME2*	*PRRT2*	*ST3GAL5*
*ARHGEF9*	*CLCN2*	*EEF1A2*	*GRIN2A*	*MECP2*	*RBFOX1*	*STRADA*
*ARX*	*CLCN4*	*EFHC1*	*GRIN2B*	*MEF2C*	*RBFOX2*	*STXBP1*
*ASAH1*	*CLN3*	*ELP4*	*HAX1*	*MFSD8*	*RBFOX3*	*SYNGAP1*
*ATP13A4*	*CLN5*	*EPHB2*	*HDAC4*	*MTHFR*	*RELN*	*SYNJ1*
*ATP1A2*	*CLN6*	*ERBB4*	*HEXA*	*MTOR*	*RYR3*	*SZT2*
*ATP1A3*	*CLN8*	*FASN*	*HEXB*	*NDE1*	*SCN1A*	*TBC1D24*
*ATP6AP2*	*CNTN5*	*FLNA*	*HNRNPH1*	*NEDD4L*	*SCN1B*	*TCF4*
*ATP7A*	*CNTNAP2*	*FOLR1*	*HNRNPU*	*NID2*	*SCN2A*	*TNK2*
*BRAF*	*COX6B1*	*FOXG1*	*IQSEC2*	*NRXN1*	*SCN8A*	*TPP1*
*BSN*	*CSTB*	*FOXP2*	*KCNB1*	*PAFAH1B1*	*SHANK3*	*TSC1*
*CACNA1A*	*CTNNA3*	*GABBR2*	*KCNH5*	*PCDH19*	*SLC13A5*	*TSC2*
*CACNA1H*	*CTSD*	*GABRA1*	*KCNMA1*	*PDHA1*	*SLC19A3*	*TUBA1A*
*CACNB4*	*CYB5R3*	*GABRA6*	*KCNQ2*	*PIGA*	*SLC1A3*	*UBE3A*
*CASR*	*DBH*	*GABRB2*	*KCNQ3*	*PIGV*	*SLC25A22*	*VRK2*
*CDH13*	*DCX*	*GABRB3*	*KCNT1*	*PLCB1*	*SLC2A1*	*WDR45*
*CDH9*	*DEPDC5*	*GABRD*	*LGI1*	*PNKD*	*SLC35A2*	*ZEB2*
*CDKL5*	*DGKD*	*GABRG2*	*LIAS*	*PNKP*	*SLC46A1*	

#### Data analysis and pathogenicity of candidate variants

After sequencing, raw data were saved in FASTQ format. Illumina sequencing adapters and low quality reads (< 80 bp) were filtered by Cutadapt [14]. Clean reads were aligned to UCSC hg19 human reference genome using the Burrows-Wheeler Alignment [15] tool. Duplicated reads were removed using Picard (http://broadinstitute.github.io/picard). Insertions, deletions and SNP variants were detected and filtered using the Genome Analysis Toolkit [16]. Then the identified variants were annotated using ANNOVAR [17] and associated with the following databases: 1000 genomes, Exome Aggregation Consortium, The Human Gene Mutation Database, and predicted by Mutation Taster (MT) [18], Sorting Intolerant From Tolerant (SIFT) [19], PolyPhen-2 (PP2) [20] and Genomic Evolutionary Rate Profiling (GERP++) [21, 22]. Splice-site were predicted by Human Splicing Finder [23]. All variants identified by the Illumina HiSeq X Ten sequencer were confirmed by Sanger sequencing. The pathogenicity of mutations was assessed in accordance with American College of Medical Genetics and Genomics guideline (ACMG) [9–11].

### Statistical analysis

Statistical analysis was performed using SPSS19. The yields of deleterious variants in patients with different onset age or family history were compared using the chi-squared test.

## Results

In the current study, we recruited 172 epilepsy pediatric patients, including 23 with Dravet syndrome, ten with Ohtahara syndrome, two with Ohtahara syndrome evolving to West syndrome, ten with West syndrome, two with West syndrome evolving to Lennox-Gastaut syndrome, five with Lennox-Gastaut syndrome, four with Doose syndrome, two with epilepsy of infancy with migrating focal seizures, two with epileptic encephalopathy with continuous spike and wave during sleep, and one each with temporal lobe epilepsy, early myoclonic encephalopathy, Landau-Kleffner syndrome, and glucose transporter type 1 deficiency syndrome. Three patients had Rett syndrome, five had tuberous sclerosis complex, and one had Sturge-Weber syndrome. Forty-two patients were diagnosed as unclassified epileptic encephalopathy and 57 patients were diagnosed as unclassified refractory epilepsy due to nonspecific manifestations (Table 2).

**Table 2:** Clinical diagnosis in 172 refractory epilepsy and their pathogenic or likely pathogenic mutations

Clinical diagnosis	Cases	P/LP mutations	P/LP gene(recurrent no.)
DS	23	16	*SCN1A* (16)
OS	10	2	*KCNQ2* (1), *SCN2A* (1)
OS-WS	2	1	*STXBP1* (1)
WS	10	4	*STXBP1* (1), *KCNT1* (1), *CDKL5* (1), *ADSL* (1)
WS-LGS	2	–	*–*
LGS	5	–	*–*
EIMFS	2	–	*–*
ECSWS	2	–	*–*
EME	1	–	*–*
LKS	1	–	*–*
UEE	42	8	*CACNA1A* (1), *GABRA1* (1), *GABRB3* (1), *SCN8A* (2), *IQSEC2* (1), *PCDH19* (1), *CHD2* (1)
Doose	4	1	*SYNGAP1* (1)
TLE	1	–	*–*
GLUT1-DS	1	1	*SLC2A1* (1)
Rett	3	1	*MECP2* (1)
TSC	5	5	*TSC2* (5)
SWS	1	–	*–*
UE	57	4	*VRK2* (1), *ATP1A2* (1), *TSC* (1), *SLC9A6* (1)
Total	172	43	–

*P* pathogenic, *LP* likely pathogenic, *DS* Dravet syndrome, *OS* Ohtahara syndrome, *OS-WS* Ohtahara syndrome evolves to West syndrome, *WS* West syndrome, *WS-LGS* West syndrome evolves to Lennox-Gastaut syndrome, *LGS* Lennox-Gastaut syndrome, *Doose* Doose syndrome, *ECSWS* epileptic encephalopathy with continuous spike and wave during sleep, *EIMFS* epilepsy of infancy with migrating focal seizures, *TLE* temporal lobe epilepsy, *EME* early myoclonic encephalopathy, *LKS* Landau-Kleffner syndromes, *UEE* unclassified epileptic encephalopathy, *GLUT1-DS* glucose transporter type 1 deficiency syndrome. *Rett* Rett syndrome, *TSC* tuberous sclerosis complex, *SWS* Sturge-Weber syndrome, *UE* unclassified refractory epilepsy

One hundred fifty-three epilepsy-related genes were selected for sequencing in all patients. The expression pattern of the targeted 153 genes across tissues were analyzed and classified according to the National Center for Biotechnology Information (NCBI, https://www.ncbi.nlm.nih.gov) and The Human Protein Atlas (https://www.proteinatlas.org) database (Additional file 1: Table S1). In our 153-gene panel, 51 genes show elevated expression, 14 genes have low expression, and 88 of them exhibit medium levels of expression in brain. The 14 low-expression genes have been associated with epilepsy, including: *ARG1* [24–27], *ARHGEF15* [28], *CASR* [29, 30], *CHRNA2* [31], *DBH* [32–34], *DIAPH3* [35], *FOLR1* [36, 37], *GABRA6* [38, 39], *GLRA1* [40, 41], *NID2* [42, 43], *PROC* [44], *SLC13A5* [45, 46], *SLC19A3* [47], *SRPX2* [48]. Specifically, among 51 elevated genes in brain, 4 genes (*GABRG2*, *GABBR2*, *GABRA1*, *GRIN1*) show restricted brain expression.

The DNA samples of patients were analyzed by using NGS and the variants were validated by Sanger Sequencing. For the samples subjected to targeted sequencing, the quality assurance (QA) /quality control (QC) file are provided in Additional file 1: Table S2.

**Fig. 1:**
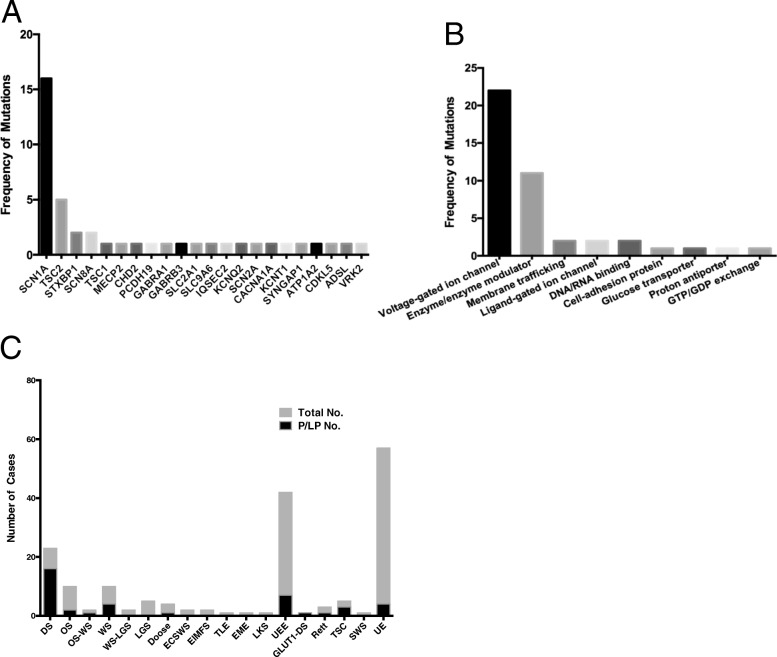
Mutated pathogenic or likely pathogenic genes in 172 refractory epilepsy children. **a** The frequency of mutated genes scored as pathogenic or likely pathogenic adhered to ACMG; **b** Functional classification of the mutated pathogenic or likely pathogenic genes; **c** The yield of pathogenic and likely pathogenic variants according to the electroclinical phenotype. Abbreviations: DS, Dravet syndrome; OS, Ohtahara syndrome; OS-WS, Ohtahara syndrome evolves to West syndrome; WS, West syndrome; WS-LGS, West syndrome evolves to Lennox-Gastaut syndrome; LGS, Lennox-Gastaut syndrome; Doose, Doose syndrome; ECSWS, epileptic encephalopathy with continuous spike and wave during sleep; EIMFS, epilepsy of infancy with migrating focal seizures; TLE, temporal lobe epilepsy; EME, early myoclonic encephalopathy; LKS, Landau-Kleffner syndromes; UEE, unclassified epileptic encephalopathy; GLUT1-DS, glucose transporter type 1 deficiency syndrome; Rett, Rett syndrome; TSC, tuberous sclerosis complex; SWS, Sturge-Weber syndrome; UE, unclassified refractory epilepsy

After sequencing the 153 epilepsy genes, we identified 43 deleterious variants in 23.3% patients (40 of 172), with three children harbouring more than one deleterious variant. Our results were similar to previous reports, with diagnostic yields ranging between 10% and 48.5% [49–56]. There were 60.5% (26/43) novel deleterious variants found in our study. A total of 43 variants in 22 genes were scored as pathogenic or likely pathogenic, including *SCN1A* (16), *TSC2* (5), *STXBP1* (2), *SCN8A* (2), *TSC1*(1), *MECP2* (1), *CHD2* (1), *PCDH19* (1), *GABRA1* (1), *GABRB3* (1), *SLC2A1* (1), *SLC9A6* (1), *IQSEC2* (1), *KCNQ2* (1), *SCN2A* (1), *CACNA1A* (1), *KCNT1* (1), *SYNGAP1* (1), *ATP1A2* (1), *CDKL5* (1), *ADSL* (1), *VRK2* (1) (Fig. 1a)*.* Among these 43 pathogenic or likely pathogenic variants, there were 18 (41.9%) missense mutations, 3 (7%) splice site mutations, 11 (25.6%) nonsense mutations, 10 (23.3%) frame-shifts, and 1 (2.3%) deletion mutations (Fig. 1a, Table 3).

**Table 3:** Pathogenic and likely pathogenic mutations adhered to ACMG guidelines in 172 refractory epilepsy children

Case code	Gene	Gene location	Transcript	cDNA change	Protein change	SIFT	PP2	MT	HSF	GERP++	MAF-ExAC	MAF-KG	Parental Origin	ACMG scoring	ACMG pathogenicity	Diagosis
13	*SCN1A*	chr2–166,901,702	NM_006920	c.1513A > T	p.K505X	–	–	A	–	6.17 (C)	–	–	*De novo*	PVS1 + PS2 + PM2	LP	DS
23	*SCN1A*	chr2–166,854,657 166,854,660 ^a^ [101]	NM_006920	c.4331_4334del	p.E1444fs	–	–	–	–	–	–	–	*De novo*	PVS1 + PS1 + PS2 + PM2	P	DS
26	*SCN1A*	chr2–166,870,270	NM_001165963	c.3689T>C	p.L1230P	D	D	D	–	5.28 (C)	–	–	*De novo*	PS2 + PM1 + PM2 + PP3	LP	DS
35	*SCN1A*	chr2–166,900,287 166,900,288	NM_001165963	c.1934_1935del	p.V645fs	–	–	–	–	–	–	–	*De novo*	PVS1 + PS2 + PM2	P	DS
38	*SCN1A*	chr2–166,859,121	NM_006920	c.G4112T	p.G1371V	D	P	D	–	5.54 (C)	–	–	*De novo*	PS2 + PM2	LP	DS
53	*SCN1A*	chr2–166,894,306 166,894,337	NM_001165963	c.2895_2926del	p.Q965fs	–	–	–	–	–	–	–	Unknown	PVS1 + PM2	LP	DS
56	*SCN1A*	chr2–166,908,355 ^a^ [102]	NM_006920	c.838T > C	p.W280R	D	D	D	–	5.41 (C)	–	–	*De novo*	PS1 + PS2 + PM2 + PP3	P	DS
65	*SCN1A*	chr2–166,850,927	NM_006920	c.4549-1G > C	splicing	–	–	D	+	5.76 (C)	–	–	*De novo*	PVS1 + PS2 + PM2	P	DS
115	*SCN1A*	chr2–166,848,614	NM_006920	c.5138C > A	p.A1713D	D	D	D	–	5.8 (C)	–	–	*De novo*	PS2 + PM2 + PP3	LP	DS
124	*SCN1A*	chr2–166,848,438 ^a^ [103]	NM_006920	c.5314G > A	p.A1772T	D	D	D	–	5.69 (C)	–	–	*De novo*	PS1 + PS2 + PM2 + PP3	P	DS
130	*SCN1A*	chr2–166,854,634 166,854,639 ^a^ [101]	NM_006920	c.4352_4356del	p.Y1451Cfs*22	–	–	–	–	–	–	–	*De novo*	PVS1 + PS1 + PS2 + PM2	P	DS
140	*SCN1A*	chr2–166,911,210 166,911,211	NM_006920	c.539delT	p.L180X	–	–	–	–	–	–	–	*De novo*	PVS1 + PS2 + PM2	P	DS
148	*SCN1A*	chr2–166,901,579	NM_001165963	c.1636G > T	p.E546X	–	–	A	–	6.17 (C)	–	–	Unknown	PVS1 + PM2	LP	DS
149	*SCN1A*	chr2–166,894,430 ^a^ [104]	NM_006920	c.2769G > A	p.M923I	D	D	D	–	5.18 (C)	–	–	Paternal	PS1 + PM2 + PP3	LP	DS
162	*SCN1A*	chr2–166,848,043 166,848,045	NM_001165963	c.5740_5742del	p.1914_1914del	–	–	–	–	–	–	–	*De novo*	PS2 + PM2 + PM4	LP	DS
172	*SCN1A*	chr2–166,903,330	NM_006920	c.1327G > T	p.E443X	–	–	A	–	5.31 (C)	–	–	*De novo*	PVS1 + PS2 + PM2	P	DS
93	*SCN2A*	chr2–166,243,416	NM_001040142	c.4712T > C	p.I1571T	D	D	D	–	5.17 (C)	–	–	*De novo*	PS2 + PM1 + PM2 + PP3	LP	OS
55	*KCNQ2*	chr20–62,073,781 ^a^ [105]	NM_172107	c.794C > T	p.A265V	D	P	D	–	3.38 (C)	–	–	*De novo*	PS1 + PS2 + PM2	P	OS
90	*STXBP1*	chr9–130,423,419 ^a^ [53]	NM_003165	c.364C > T	p.R122X	–	–	A	–	4.92 (C)	–	–	Unknown	PVS1 + PS1 + PM2	P	OS-WS
52	*ADSL*	chr22–40,745,935	NM_000026	c.253C > T	p.R85X	–	–	A	–	5.59 (C)	–	–	Maternal	PVS1 + PM2	LP	WS
		chr22–40,742,633 [58]	NM_000026	c.71C > T	p.P24L	T	B	D	–	0.153 (N)	–	–	Paternal	PM2	UC	
89	*KCNT1*	chr9–138,651,532 ^a^ [106]	NM_020822	c.862G > A	p.G288S	T	D	D	–	5.05 (C)	–	–	*De novo*	PS1 + PS2 + PM1 + PM2	P	WS
104	*CDKL5*	chrX-18,593,592 18,593,593	NM_003159	c.265delT	p.F89Lfs^*^24	–	–	–	–	–	–	–	*De novo*	PVS1 + PS2 + PM2	P	WS
151	*STXBP1*	chr9–130,428,529	NM_003165	c.748C > T	p.Q250X	–	–	A	–	5.72 (C)	–	–	*De novo*	PVS1 + PS2 + PM2	P	WS
29	*SYNGAP1*	chr6–33,393,659 33,393,662	NM_006772	c.274_277del	p.G92fs	–	–	–	–	–	–	–	*De novo*	PVS1 + PS2 + PM2	P	Doose
164	*SLC2A1*	chr1–43,396,517	NM_006516	c.296T > G	p.M99R	D	B	D	–	5.51 (C)	–	–	*De novo*	PS2 + PM2	LP	GLUT1-DS
30	*MECP2*	chrX-153,296,516 ^a^ [63]	NM_001110792	c.799C > T	p.R267X	–	–	A	–	3.55 (C)	–	–	*De novo*	PVS1 + PS1 + PS2 + PM2	P	Rett
32	*TSC2*	chr16–2,126,095 ^a^ [91]	NM_000548	c.2666C > T	p.A889V	D	D	D	–	5.09 (C)	–	–	Paternal	PS1 + PM2 + PP3	LP	TSC
94	*TSC2*	chr16–2,130,180 ^a^ [107]	NM_000548	c.3412C > T	p.R1138X	–	–	A	–	4.74 (C)	–	–	*De novo*	PVS1 + PS1 + PS2 + PM2	P	TSC
	*TSC2*	chr16–2,130,366 ^a^ [66]	NM_000548	c.3598C > T	p.R1200W	D	D	D	–	4.74 (C)	–	–	*De novo*	PS1 + PS2 + PM2 + PP3	P	
98	*TSC2*	chr16–2,138,467	NM_001077183	c.5079C > G	p.Y1693X	–	–	D	–	0.137 (N)	–	–	Paternal	PVS1 + PM2	LP	TSC
	*TSC2*	chr16–2,138,465 2,138,466	NM_001077183	c.5077delT	p.Y1693fs	–	–	–	–	–	–	–	Paternal	PVS1 + PM2	LP	
7	*SCN8A*	chr12–52,184,209 ^a^ [108]	NM_001177984	c.4324G > A	p.E1442K	D	D	D	–	4.68 (C)	–	–	Paternal	PS1 + PM2 + PP3	LP	UEE
	*IQSEC2*	chrX-53,263,621 53,263,622	NM_001111125	c.4246_4247insG	p.S1416fs	–	–	–	–	–	–	–	*De novo*	PVS1 + PS2 + PM2	P	
63	*CACNA1A*	chr19–13,566,019 ^a^ [109]	NM_001127221	c.301G > C	p.E101Q	D	D	D	–	5.01 (C)	–	–	*De novo*	PS1 + PS2 + PM1 + PM2 + PP3	P	UEE
66	*SCN8A*	chr12–52,200,885 ^a^ [110]	NM_001177984	c.5492G > A	p.R1831Q	D	D	D	–	4.91 (C)	–	–	*De novo*	PS1 + PS2 + PM2 + PP3	P	UEE
69	*PCDH19*	chrX-99,551,873 99,551,874	NM_001184880	c.2849-1G > −	splicing	–	–	–	+	–	–	–	Unknown	PVS1 + PM2	LP	UEE
157	*GABRB3*	chr15–26,812,802 ^a^ [111]	NM_021912	c.761C > T	p.S254F	D	D	D	–	6.06 (C)	–	–	*De novo*	PS1 + PS2 + PM1 + PM2 + PP3	P	UEE
160	*GABRA1*	chr5–161,309,645 ^a^ [112]	NM_001127648	c.641G > A	p.R214H	D	D	D	–	5.34 (C)	–	–	*De novo*	PS1 + PS2 + PM1 + PM2 + PP3	P	UEE
54	*CHD2*	chr15–93,540,231	NM_001271	c.3640G > T	p.G1214X	–	–	A	–	5.64 (C)	–	–	*De novo*	PVS1 + PS2 + PM2	P	UEE
40	*VRK2*	chr2–58,312,086	NM_001130483	c.C256 + 1G > A	splicing	–	–	D	+	5.86 (C)	–	–	Unknown	PVS1 + PM2	LP	UE
44	*ATP1A2*	chr1–160,098,521	NM_000702	c.1097G > T	p.G366V	D	D	D	–	4.77 (C)	–	–	*De novo*	PS2 + PM1 + PM2 + PP3	LP	UE
68	*TSC1*	chr9–135,772,854	NM_000368	c.2768_2769insC	p.L924Ffs^*^26	–	–	–	–	–	–	–	*De novo*	PVS1 + PS2 + PM2	P	UE
79	*SLC9A6*	chrX-135,080,322 135,080,336	NM_001042537	c.582_595del	p.Y194fs	–	–	–	–	–	–	–	*De novo*	PVS1 + PS2 + PM2	P	UE

*Abbreviations*: *M* male, *F* female, *m* month, *y* year, *SIFT* Sorts intolerant from tolerant (D, damaging; T, tolerant), *PP2*, polymorphism phenotyping v2 (D, damaging; P, possible damaging; B, benign), *MT* mutation taster (D, disease causing; A, disease causing automatic), *HSF* human splicing finder (+, altering splicing), *GERP++* genomic evolutionary rate profiling (C, conserved; N, nonconserved), *KG* 1000 Genomes project, *LP* likely pathogenic, *P* pathogenic, *DS* Dravet syndrome, *OS* Ohtahara syndrome, *OS-WS* OS syndrome evolves to West syndrome, *WS* West syndrome, *Doose* Doose syndrome, *GLUT1-DS* glucose transporter type 1 deficiency syndrome, *Rett* Rett syndrome, *TSC* tuberous sclerosis complex, *UEE* unclassified epileptic encephalopathy, *UE* unclassified refractory epilepsy

^a^ Mutations have been reported in HGMD database

More recent studies suggest that many severe epilepsy types begin in infancy or childhood, especially those with psychomotor retardation and epileptic encephalopathies are often due to *de novo* mutations [30, 31]. In our study, 32/43 (74.4%) pathogenic or likely pathogenic variants were *de novo*, five (11.6%) were paternal, one (2.3%) was maternal, and five (11.6%) were unknown due to blood samples from parents were unavailable (Table 3).

To further explore the genetic pathogenesis of epilepsy, we subdivided the mutated genes into nine groups according to the molecular and biological function of the gene produce. These functional groups included voltage-gated ion channels, enzyme/enzyme modulators, membrane trafficking, ligand-gated ion channels, DNA/RNA binding, cell-adhesion proteins, glucose transporter, proton antiporter, and GTP/GDP exchanges. Variants in ion channel genes (*SCN1A, SCN2A, SCN8A, CACNA1A, KCNT1, KCNQ2*) accounted for 51.2% (22/43) of the pathogenic or likely pathogenic variants. Variants in enzyme/enzyme modulator genes (*TSC1, TSC2, SYNGAP1, ATP1A2, CDKL5, ADSL, VRK2*) accounted for 25.6% (11/43) of pathogenic or likely pathogenic variants. Variants in genes encoded membrane trafficking (*STXBP1*), ligand-gated ion channels (*GABRA1, GABRB3*), DNA/RNA binding proteins (*MECP2, CHD2*) each accounted for 4.7% (2/43) (Fig. 1b). Ion channels (voltage-gated and ligand-gated) accounted for 55.8% in total, suggesting that dysfunction of ion channels plays critical roles in the pathogenesis of epilepsy.

We then analyzed the yield of the epilepsy gene panel testing based on electroclinical syndrome (Fig. 1c). The yield of deleterious variants in Dravet syndrome (69.6%, 16/23) and glucose transporter type 1 deficiency syndrome (100%, 1/1) patients was higher than that in others. Patients with onset age of seizures ≤12 months had higher yields of deleterious variants compared to those with onset age of seizures > 12 months (31/101 vs 9/71; χ2 = 7.583, df = 1, *P* = 0.006). The family history did not affect whether or not a deleterious genetic variant was identified (7/27 vs 33/145; χ2 = 0.128, df = 1, *P* = 0.804).

**Fig. 2:**
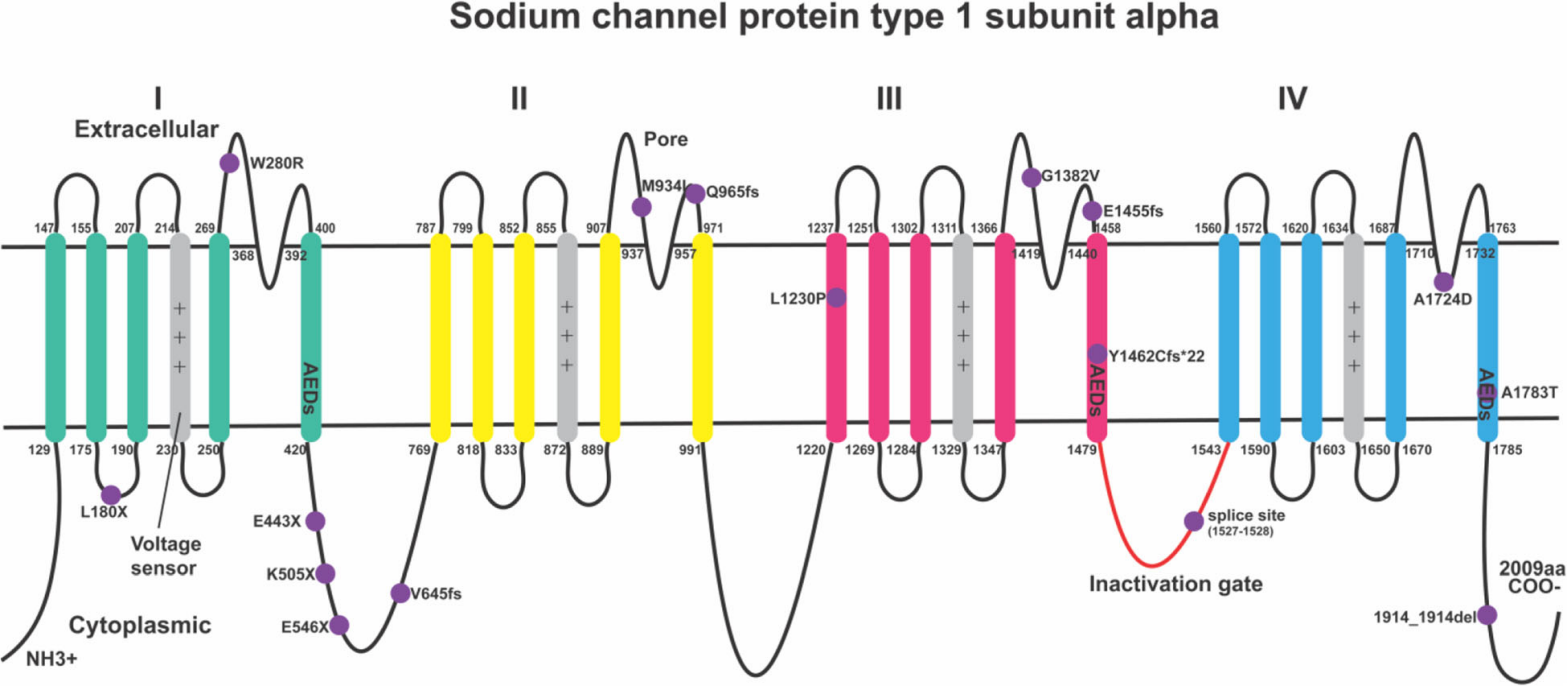
Schematic representation of the mutations in subunit alpha of sodium channel type 1 (SCN1A) in our study. SCN1A alpha unit has four domains (I–IV), each domain includes 6 transmembrane segments (S1–S6). Purple circle = mutation; AEDs, anti-epileptic drugs. The position of mutations in SCN1A is approximate and is according to reference transcript NM_001165963.

There were 16 mutations in *SCN1A* gene, of which six (37.5%) were missense mutations, one (6.25%) was a splice site mutation, four (25%) were nonsense mutations, four (25%) were frame-shifts, and one (6.25%) was deletion mutation. Thirteen of the 16 (81.3%) *SCN1A* mutations were *de novo* and 11 (68.8%) were novel. We further analysed the positions of the mutations in the affected proteins corresponding to gene mutations and found that 43.8% (7/16) of protein changes are in the intracellular loop of sodium channel protein type 1 subunit alpha, 31.3% (5/16) are in the extracellular loop, 18.8% (3/16) are in the transmembrane region, and 6.25% (1/16) are in the pore forming area (Fig. 2).

There has been a marked increase in genetic diagnoses of a number of key childhood-onset epilepsy syndromes, such as Dravet syndrome, which has been mainly linked to *SCN1A* [17]. In our 16 patients diagnosed as Dravet syndrome with pathogenic or likely pathogenic variants, all identified mutations were in the *SCN1A* gene. These 16 Dravet syndrome patients had typical manifestations: onset between 3 to 8 months of age, fever-sensitive, multiple seizure types, and developmental delay after seizure onset. 81.25% (13/16) *SCN1A* mutations were *de novo* in Dravet syndrome patients and one was inherited from the father who had a history of febrile seizures (FS). 12.5% (2/16) *SCN1A* mutations were unknown. Pathogenic and likely pathogenic mutations each accounted for 50% (Table 4). 50% (8/16) of the Dravet syndrome variants cause nonsense or frameshift mutations that result in truncated proteins, which was consistent with a previous study [57]. We evaluated whether different seizure types, family history, abnormal brain MRI, or developmental delay were associated with specific *SCN1A* mutation types or locations within the gene. We did not detect any bias towards particular regions of the gene or in the type of mutation, although our small sample size did not provide substantial power (Additional file 1: Tables S3 and S4).

**Table 4:** Clinical features in DS patients

Case code	Gender/age	Diagosis	Age of onset	Seizure types	EEG	Brain MRI/CT	Developmental delay	Gene	cDNA change	Protein change	Parental Origin	ACMG pathogenicity
13	F/2y6m	DS	3m	FS, FoS, Myo	FSW	Normal	Yes	*SCN1A*	c.1513A > T	p.K505X	*De novo*	LP
23	F/3y	DS	7m	FS, FoS (A), Myo, FBTC	Multi. FD	Underdeveloped myelin	Yes	*SCN1A*	c.4331_4334del	p.E1444fs	*De novo*	P
26	F/5y11m	DS	5m	FS, SE, FoS (A), Myo, FBTC	FSW	Normal	Yes	*SCN1A*	c.3689T>C	p.L1230P	*De novo*	LP
35	M/4y	DS	3m	FS, SE, GTCS, aAb	Multi. FD	Normal	Yes	*SCN1A*	c.1934_1935del	p.V645fs	*De novo*	P
38	F/1y6m	DS	4m	FS, SE, Myo	FSW	Nonspecific	Yes	*SCN1A*	c.G4112T	p.G1371V	*De novo*	LP
53	M/5y	DS	7m	FS, aAb, Myo, Fos (I)	Multi. FD	Normal	Yes	*SCN1A*	c.2895_2926del	p.Q965fs	Unknown	LP
56	F/3y6m	DS	5m	FS, Myo, GTCS, SE, FoS (A), aAb	Multi. FD	Nonspecific	Yes	*SCN1A*	c.838T > C	p.W280R	*De novo*	P
65	M/2y4m	DS	5m	FS, SE, FoS (A)	FSW	Normal	Yes	*SCN1A*	c.4549-1G > C	splicing	*De novo*	P
115	M/2y1m	DS	8m	FS, FoS (I), FoS (hemi clonic), GTCS	FSW	Enlargement of the subarachnoid space in front of left temporal lobe	Yes	*SCN1A*	c.5138C > A	p.A1713D	*De novo*	LP
124	M/3y	DS	5m	FS, FoS (A), FBTC	FSW	Nonspecific	Yes	*SCN1A*	c.5314G > A	p.A1772T	*De novo*	P
130	F/11y	DS	6m	FS, FoS (A), aAb, Myo, GTCS	Multi. FD	Normal	Yes	*SCN1A*	c.4352_4356del	p.Y1451Cfs*22	*De novo*	P
140	F/1y9m	DS	3m	FS, GTCS, C, FoS (I)	FSW	Normal	Yes	*SCN1A*	c.539delT	p.L180X	*De novo*	P
148	F/6y8m	DS	4m	FS, GTCS, FoS, aAb	Multi. FD	Normal	Yes	*SCN1A*	c.1636G > T	p.E546X	Unknown	LP
149	M/3y6m	DS	4m	FS, FoS (A), Myo, GTCS	Multi. FD, GSW	Normal	Yes	*SCN1A*	c.2769G > A	p.M923I	Paternal (FS)	LP
162	M/4y	DS	5m	FS, FoS (A), Myo, FBTC	Multi. FD	Normal	Yes	*SCN1A*	c.5740_5742del	p.1914_1914del	*De novo*	LP
172	F/8y	DS	5m	FS, aAb, Myo, FBTC	Multi. FD, GSW, GPSW	Normal	Yes	*SCN1A*	c.1327G > T	p.E443X	*De novo*	P

*Abbreviations*: *M* male, *F* female, *m* month, *y* year, *P* pathogenic, *LP* likely pathogenic, *UC* uncertain, *DS* Dravet syndrome, *FS* febrile seizures, *SE* status epilepticus, *FoS* focal seizures, *FoS (I)* focal seizures (impaired awareness), *FoS (A)* focal seizures (aware), *FBTC* focal to bilateral tonic-clonic, *Myo* myoclonic, *aAb* atypical absence, *GTCS* generalized tonic-clonic seizures, *FSW* focal spike wave, *Multi. FD* multifocal discharges, *GSW* generalized spike-wave, *GPSW* generalized polyspike-wave

Twelve patients presented typical manifestation of Ohtahara syndrome: onset age within postnatal 30 days, tonic spasms, burst suppression EEG and developmental delay. Pathogenic or likely pathogenic variants in Ohtahara syndrome were in the *KCNQ2* (1), *STXBP1* (1), *SCN2A* (1) genes. The nonsense mutation in *STXBP1* (c.364C > T, p.R122X) was detected in one Ohtahara syndrome patients that evolved to West syndrome. This patient had an onset age of postnatal 17 day, spasms, and burst-suppression EEG at postnatal 22 day and hypsarrhythmia EEG at 4 months (Table 5).

**Table 5:** Clinical features in OS, WS, LGS, Doose, GLUT1-DS, Rett, TSC, UEE and UE patients

Case code	Gender/age	Diagosis	Age of onset	Seizure types	EEG	Brain MRI/CT	Developmental delay	Gene	cDNA change	Protein change	Parental Origin	ACMG pathogenicity
55	M/54d	OS	1d	FoS, Tonic spasms	BS, FSW	Normal	Yes	*KCNQ2*	c.794C > T	p.A265V	*De novo*	P
93	M/40d	OS	3d	Tonic spasms	BS	Normal	Yes	*SCN2A*	c.4712T > C	p.I1571T	*De novo*	LP
90	M/2y11m	OS-WS	17d	Tonic spasms, Spa.	BS, Hypsarrhy.	Normal	Yes	*STXBP1*	c.364C > T	p.R122X	Unknown	P
52	F/1y8m	WS	2m	Spa.	Multi. FD, Hypsarrhy.	Cerebral dysplasia	Yes	*ADSL*	c.253C > T	p.R85X	Maternal	LP
								*ADSL*	c.71C > T	p.P24L	Paternal	UC
89	F/1y11m	WS	19d	FoS, Spa.	Multi. FD, Hypsarrhy.	Subdural hemorrhage	Yes	*KCNT1*	c.862G > A	p.G288S	*De novo*	P
104	F/2y10m	WS	3m7d	Spa.	Hypsarrhy., Multi.FD	Normal	Yes	*CDKL5*	c.265delT	p.F89Lfs*24	*De novo*	P
151	F/9m	WS	3m	Spa.	Hypsarrhy., Multi. FD	Enlargement of the subarachnoid space	Yes	*STXBP1*	c.748C > T	p.Q250X	*De novo*	P
29	M/5y6m	Doose	1y3m	Myo-At., Myo, aAb	Abnormal background theta, GSW, GPSW	Normal	No	*SYNGAP1*	c.274_277del	p.G92fs	*De novo*	P
164	F/6y	GLUT1-DS	2y4m	GTCS	FSW, Multi. FD	Nonspecific (Hair loss leads to bald)	No	*SLC2A1*	c.296T > G	p.M99R	*De novo*	LP
30	F/4y4m	Rett	3y2m	Fos (I), FBTC	Multi. FD	Normal	Yes	*MECP2*	c.799C > T	p.R267X	*De novo*	P
32	M/8y	TSC	1y6m	FoS (I), FBTC	Multi. FD	Multi nodules	No	*TSC2*	c.2666C > T	p.A889V	Paternal	LP
94	F/9m	TSC (WS)	3m	Spa.	Multi. FD, Hypsarrhy.	Multi nodules	Yes	*TSC2*	c.3412C > T	p.R1138X	*De novo*	P
								*TSC2*	c.3598C > T	p.R1200W	*De novo*	P
98	M/3y	TSC (WS)	4m	Spa., aAb	Multi. FD, Hypsarrhy.	Nonspecific	Yes	*TSC2*	c.5079C > G	p.Y1693X	Paternal	LP
								*TSC2*	c.5077delT	p.Y1693fs	Paternal	LP
7	M/2y	UEE (EIEE13)	6m	FoS (I), FBTC	Multi. FD	Enlargement of the subarachnoid space	Yes	*SCN8A*	c.4324G > A	p.E1442K	Paternal	LP
								*IQSEC2*	c.4246_4247insG	p.S1416fs	*De novo*	P
63	M/4y	UEE (EIEE42)	5m	FoS, GTCS	Multi. FD	Normal	Yes	*CACNA1A*	c.301G > C	p.E101Q	*De novo*	P
66	M/1y9m	UEE (EIEE13)	4m	FBTC, FoS	Multi. FD	Enlargement of the subarachnoid space	Yes	*SCN8A*	c.5492G > A	p.R1831Q	*De novo*	P
69	F/2y1m	UEE (EIEE9)	1y3m	FBTC, C, T	Multi. FD	Normal	Yes	*PCDH19*	c.2849-1G > −	splicing	Unknown	LP
157	F/2y	UEE (EIEE43)	2m	C, FoS (I)	FSW	Normal	Yes	*GABRB3*	c.761C > T	p.S254F	*De novo*	P
160	M/6y	UEE (EIEE19)	6m	FoS (I), GTCS	FSW	Normal	Yes	*GABRA1*	c.641G > A	p.R214H	*De novo*	P
54	F/7y	UEE (EEOC)	4y2m	SE, GTCS, FoS (I)	Mult. FD	Normal	Yes	*CHD2*	c.3640G > T	p.G1214X	*De novo*	P
40	F/2y11m	UE	4m	FoS	FSW	Normal	No	*VRK2*	c.C256 + 1G > A	splicing	Unknown	LP
44	F/5y6m	UE	4y	FoS (automatisms, emotional)	Multi. FD	Nodules in internal side of left anterior limb of internal capsule; caput of caudate nucleus or heterotopic gray matter	Yes	*ATP1A2*	c.1097G > T	p.G366V	*De novo*	LP
68	M/6y	UE	4y	FoS (A)	FSW	Normal	No	*TSC1*	c.2768_2769insC	p.L924Ffs*26	*De novo*	P
79	M/3y	UE	1y2m	FoS (I), FBTC	Multi. FD	Normal	Yes	*SLC9A6*	c.582_595del	p.Y194fs	*De novo*	P

*Abbreviations*: *M* male, *F* female, *m* month, *y* year, *P* pathogenic, *LP* likely pathogenic, *UC* uncertain, *OS* Ohtahara syndrome, *OS-WS* Ohtahara syndrome evolves to West syndrome, *WS* West syndrome, *Doose* Doose syndrome, *GLUT1-DS* glucose transporter type 1 deficiency syndrome, *Rett* Rett syndrome, *TSC* tuberous sclerosis complex, *UEE* unclassified epileptic encephalopathy, *UE* unclassified refractory epilepsy, *EEIE* early-infantile epileptic encephalopathies, *EEOC* childhood-onset epileptic encephalopathy, *Spa.* Spasms, *FoS* focal seizures, *FoS (I)* focal seizures (impaired awareness), *FoS (A)* focal seizures (aware), *FBTC* focal to bilateral tonic-clonic, *T* tonic, *C* clonic, *Myo* myoclonic, *aAb* atypical absence, *At.* atonic, *GTCS* generalized tonic-clonic seizures, *SE* status epilepticus, *BS burst suppression*, *Hypsarrhy.* hypsarrhythmia, *Multi. FD* multifocal discharges, *FSW* focal spike-wave, *GSW* generalized spike-wave, *GPSW* generalized polyspike-wave

West syndrome patients in our study had onset ages of seizures ranging from postnatal 19 days to 6 months. Typical clinical manifestations were all observed, including spasms, hypsarrhythmia EEG, and developmental delay. 16.7% (2/12) of the West syndrome children evolves to Lennox-Gastaut syndrome. After sequencing, we identified 4 pathogenic or likely pathogenic mutations in the following genes: *STXBP1* (1), *KCNT1* (1), *CDKL5* (1), *ADSL* (1). 75% (3/4) of these variants were *de novo*.

One of the West syndrome patients were found to carry two mutations: a nonsense *ADSL* (c.253C > T, p.R85X) mutation was scored as likely pathogenic and was inherited from her unaffected mother. Another reported missense *ADSL* (c.71C > T, p.P24L) [58] mutation which was inherited from her unaffected father were scored as uncertain pathogenicity. *ADSL* has been reported to be related to adenylosuccinate lyase deficiency, which is an autosomal recessive defect of purine metabolism [59, 60]. The patient presented with spasms 2 months after birth. Brain MRI showed cerebral dysplasia and EEG showed hypsarrhythmia and multifocal discharges. The patient also had developmental delay and lack of eye contact. A definitive diagnosis can be made with high performance liquid chromatography examination of the urine to detect the ratio of succinyladenosine and succinyl-aminoimidazole carboximide riboside, but this was not available for the patient in question. Thus, this patient was diagnosed clinically as having West syndrome.

A novel frame-shift mutation in *SYNGAP5* (c.274_277del, p.G92fs) was detected in a patient with Doose syndrome. This patient presented with myoclonic and myoclonic-astatic seizures, as well as having atypical absence seizures. *SYNGAP5* had been reported to be associated with Doose syndrome and mental retardation, autosomal dominant 5 (MRD5) [51, 61, 62]. This mutation, which is very rare, was *de novo*, and caused frameshift changes in Ras/Rap GTPase-activating protein SynGAP, was therefore scored as pathogenic (Table 5).

One glucose transporter type 1 deficiency syndrome patient presented with seizures at age 28 months. The patient has alopecia and was almost bald at 4 years old. The child did not have other abnormalities in blood tests, brain MRI, or neurological exam. Her cerebrospinal fluid glucose value was 2.04 mmol/L (blood glucose value was 7.2 mmol/L before lumbar puncture; fasting blood glucose value was 5.2 mmol/L). NGS identified a missense mutation in *SLC2A1* (c.296T > G, p.M99R). The mutation was *de novo* and novel. The patient’s parents and sister were normal, which is consistent with the sequencing results. Symptoms improved with a ketogenic diet, with seizures controlled for more than 6 months.

One *MECP2* mutation (c.799C > T, p.R267X) was detected in a girl diagnosed as Rett syndrome. The girl developed normally for the first 18 months, gradually lost speech ability while developing repetitive hand-wringing. Seizures began at age 3 years. The *MECP2* gene is located on the X-chromosome, and Rett syndrome is inherited through this gene in a dominant fashion [63]. This patient had a *de novo* *MECP2* nonsense mutation, consistent with her parents being unaffected.

40% (2/5) of tuberous sclerosis complex patients were diagnosed with West syndrome associated with tuberous sclerosis complex in our study. Tuberous sclerosis complex is closely related to the *TSC1/TSC2* genes [64–67]*.*

In our study, all of the tuberous sclerosis complex patients’ initial presentations were seizures, of which 80% (4/5) presented in the first year of life. 60% (3/5) had hypomelanotic macules and 40% (2/5) had multi nodules. One patient’s only clinical manifestation was seizures and three (60%) patients with seizures had only one major feature of tuberous sclerosis complex. After sequencing, 60% (3/5) patients were found to have deleterious *TSC1* or *TSC2* mutations.

We identified more than one *TSC1/2* mutations in 2 patients. One patient has two *TSC2* mutations inherited from his affected father. Facial angiofibromas appeared by age 3–4 years in 60% (3/5) patients in the follow-up period. Gilboa et al. [68] reported four patients with the same *TSC1* genomic deletion (9q34.13q34.2) in a family and none of them fulfilled the clinical criteria for tuberous sclerosis complex. In our study, one patient with pathogenic *TSC1* (c.2768_2769insC, p.L924Ffs*26) mutation presented with focal seizures beginning at age four. There were two hypopigmented macules on the patient’s abdomen. The brain MRI results were normal and there are no other features of tuberous sclerosis complex. This *de novo* mutation causes a frame-shift in hamartin and has not been reported previously. Thus, this patient was considered to have unclassified refractory epilepsy.

One unclassified epileptic encephalopathy patient had two deleterious mutations: *SCN8A* inherited from his affected father (c.4324G > A, p.E1442K) and *IQSEC2* (c.4246_4247insG, p.S1416fs). Early-infantile epileptic encephalopathies (EIEE) caused by *SCN8A* mutations are designated as EIEE13 (OMIM #614558) [69]. The missense mutation in *SCN8A* is very rare in the general population, and had been previously predicted to be damaging by SIFT, MT and PP2. *IQSEC2* is an X-linked gene that has been reported to be related to intellectual disability and epilepsy, and it encodes the IQ motif and SEC7 domain-containing protein 2 [70]. The identified novel *IQSEC2* mutation was *de novo* and was scored as being pathogenic.

Other pathogenic or likely pathogenic mutations found in patients with unclassified epileptic encephalopathy included *CACNA1A, GABRA1, GABRB3, PCDH19,* and *CHD2.* Epileptic encephalopathies with the above mutations had been designated as EIEE42, EIEE19, EIEE43, EIEE9 and EEOC (childhood-onset epileptic encephalopathy) according to Online Mendelian Inheritance in Man (OMIM). Other deleterious variants found in patients with unclassified refractory epilepsy were in *VRK2, ATP1A2,* and *SLC9A6.* Taking these unclassified epileptic encephalopathies and unclassified refractory epilepsy patients’ clinical manifestations into consideration, we found that all patients with deleterious mutations in genes encoding ion channels (*SCN8A, CACNA1A, GABRB3, GABRA1*) had similar clinical symptoms: onset age of seizures within the first year, epileptic encephalopathy and developmental delay. In contrast, patients with mutations in *VRK2, ATP1A2,* and *SLC9A6*, had relatively later onset age of seizures.

We then assessed the clinical benefit of genetic testing in those patients with identified deleterious variants. NGS helped with the diagnosis (*n* = 8), medication selection (*n* = 18), reproductive planning (*n* = 4), and treatment planning (*n* = 1). The finding of the *SLC2A1* variant in Case 164 prompted other tests such as cerebrospinal fluid (CSF) glucose that were clinically useful. Identification of deleterious *SCN1A* mutations in five young infants with clinically suspected Dravet syndrome helped early diagnosis (Case 13, 38, 65, 115, 140) and led to the discontinuation of oxcarbazepine (Case 13) that exacerbated seizures. Identification of *SCN1A* mutations in other Dravet syndrome patients helped to avoid sodium channel blockers such as oxcarbazepine, carbamazepine and lamotrigine. Among the four Dravet syndrome patients who responded to anticonvulsants (Case 13, 26, 149, 172), 75% (3/4) of them were prescribed sodium valproate or clonazepam suggesting that these medications may be effective in Dravet syndrome. The finding of the *TSC2* variants in Cases 94 and 98 helped early diagnosis and Case 32 experienced remission with administration of rapamune. Identification of *TSC1* prompted clinical surveillance for tuberous sclerosis complex in Case 68. The findings of patients with deleterious variants in *TSC2* (Case 32, 98), *SCN8A* (Case 7), *SCN1A* (Case 149), *ADSL* (Case 52) which were inherited, helped in prenatal counselling (Table 6).

**Table 6:** Clinical benefits after molecular diagnosis

Clinical benefits		Effects (Case details)
Diagnosis	*SLC2A1* (GLUT1-DS)	Definitive diagnosis (Case 164)
	*SCN1A* (DS)	Definitive diagnosis (Case 13, 38, 65, 115, 140)
	*TSC2* (TSC)	Definitive diagnosis (Case 94, 98)
Management implications	*SLC2A1,* using KD	Controlled (Case 164, KD)
	*SCN1A*, stopping OXC	Remitted (Case 13, VPA, TPM,10–20 / month)
	*SCN1A*, avoiding OXC, CBZ, and LTG	Remitted (Case 23, VPA, TPM, seizure-free for 5 months; Case 26, LEV, TPM, CZP, seizure-free for 6 months; Case 149, VPA, TPM, LEV, CZP, seizure-free for 4 months; Case 172, VPA, TPM, CZP, seizure-free for 1 year)
		Uncontrolled (Case 35, 38, 53, 56, 65, 115, 124, 130, 140, 148, 162)
	*TSC2,* using rapamune	Remitted (Case 32, seizure-free for 7 months)
Long-term follow up	*TSC1* (risk of TSC)	Case 68
Reproductive planning	Suggesting the family conduct genetic counseling	*TSC2* (Case 32, 98), *SCN8A* (Case 7), *SCN1A* (Case 149), *ADSL* (Case 52)

*Abbreviations*: *DS* Dravet syndrome, *GLUT1-DS* glucose transporter type 1 deficiency syndrome, *Rett* Rett syndrome, *TSC* tuberous sclerosis complex, *KD* ketogenic diet, *OXC* oxcarbazepine, *CBZ* carbamazepine, *LTG* lamotrigine, *VPA* sodium valproate, *TPM* topiramate, *LEV* levetiracetam, *CZP* clonazepam

## Discussion

Epilepsy is highly heterogeneous and can be primarily genetic in origin, or be secondary to structural or metabolic disorders of the central nervous system [71, 72]. To date, over 500 genes have been implicated in epilepsy [73–76]. However, the overlapping clinical features of different epilepsy syndromes and non-specific phenotypes can hamper clinical and genetic diagnosis [53]. The correct genetic diagnosis can help to guide treatment and prognosis. In addition to genetic origins, pediatric epilepsy may also arise from epigenetic mechanisms mediating gene-environment interactions during neurodevelopment. In this study, we used NGS to investigate 153 epilepsy related genes in a cohort of 172 refractory epilepsy children.

Approximately one quarter of genes identified in epilepsy encode ion channel proteins, including voltage-gated channels (Na^+^, K^+^, Ca2^+^ channels and hyperpolarization-activated cyclic nucleotide-gated channels) and ligand-gated ion channels (N-Methyl-D-Aspartate receptors, Gamma-aminobutyric acid receptors and Nicotinic Acetylcholine receptors) [77]. The genes that encode ion channels and are relevant to epilepsy include *SCN1A, SCN1B, SCN2A, SCN8A, KCNA1, KCNA2, KCNB1, KCNC1, KCNMA1, KCNQ2, KCNQ3, KCNT1, KCTD7, HCN1, CACAN1A, CACNA1H, GRIN1, GRIN2A, GRIN2B, GRIN2D, GABRA1, GABRB3, GABRG2, CHRNA2, CHRNA4, CHRNB2.* In our study, 51.2% pathogenic or likely pathogenic variants were found in voltage-gated ion channels and 4.7% were found in ligand-gated ion channels. Thus, we further confirmed that ion channels play an important role in the pathogenesis of epilepsy.

An *SCN1A* mutation was first discovered in epilepsy in 2000 [72], and now hundreds of new *SCN1A* mutations have been described in epilepsy patients, making it the most common epilepsy-related gene [78]. In our study, we found *SCN1A* mutations in 16/44 deleterious variants, making it the most common gene to show variation in our study. *SCN1A* encodes the Nav1.1 pore-forming α-subunit, expressed mainly in inhibitory GABAergic neurons. The α-subunit comprises four homologous domains (I–IV), forming a tetrameric structure. Each domain is composed of six transmembrane segments (S1–S6) [77]. The S4, voltage-sensing segment has multiple positively charged amino acids. The intracellular loop between III and IV domain functions as the inactivation gate. The α-subunit is usually associated with two β-subunits that influence α-subunit localization and function [77]. Among α-subunit of sodium channel genetic variants in our study, 43.8% (7/16) are within the intracellular loop, 31.3% (5/16) in the extracellular loop, 18.8% (3/16) in the transmembrane area, and 6.25% (1/16) in the pore forming area. All the extracellular mutations are between S5 and S6, which is very close to the pore forming area. These variants may influence the initiation and propagation of action potentials, making these inhibitory GABAergic neurons less excitable. Some antiepileptic drugs (AEDs) bind to the inner cavity of the pore of the sodium channel (IS6, IIIS6 and IVS6) [77, 79]. The pore forming area or internal/external loop could be promising targets for new seizure prophylaxis medications.

Patients harboring *SCN1A* mutations can have with Dravet syndrome or generalized epilepsy with febrile seizures plus. One Dravet syndrome patient inherited the *SCN1A* mutations from his father only had febrile seizures. This could be due to somatic mosaicism [72, 80, 81]. A Dravet syndrome mouse model (Nav1.1 knockout-based) responded well to stiripentol and clobazam, which are commonly used to treat Dravet syndrome [82–85]. One of the patients in our study was treated with oxcarbazepine, which blocks sodium channels and worsened seizures, before the diagnosis of Dravet syndrome was made. This case illustrates the importance of correct molecular diagnosis in selecting the best anticonvulsant.

Approximately half of Ohtahara syndrome patients with *STXBP1* mutations evolve to West syndrome [86]. In our study, there was one such patient with a nonsense mutation in *STXBP1*, suggesting that this gene could play a role in the etiology of West syndrome. Our findings also suggest that *STXBP1* is related to both Ohtahara syndrome and West syndrome.

*KCNT1* is associated with epilepsy of infancy with migrating focal seizures, autosomal dominant nocturnal frontal lobe epilepsy, and other types of early onset epileptic encephalopathies [87–89]. Ohba et al. [88] found 11 *KCNT1* mutations in a total of 362 epilepsy patients: 9/18 epilepsy of infancy with migrating focal seizures cases (50%), 1/180 West syndrome cases (0.56%), and 1/66 unclassified early onset epileptic encephalopathy cases (1.52%), suggesting that *KCNT1* may be a causal gene for West syndrome. In our study, one *KCNT1* (c.862G > A, p.G288S) mutation was found in a patient diagnosed as West syndrome.

Genetic studies of neuropsychiatric disease have led to the discovery of molecular etiology and pathophysiology. For example, most cases of Rett syndrome are now known to arise from mutations in the *MECP2* gene, which codes for a methyl-CpG-binding protein 2 [90]. Another example is glucose transporter type 1 deficiency syndrome, which has been attributed to variants in *SLC2A1, SLC2A2,* and *GLUT1.* In our study, the glucose transporter type 1 deficiency syndrome patient did not have cerebrospinal fluid analysis as part of their diagnostic work-up until the genetic data suggested the diagnosis. This example illustrates the utility of NGS in clinical scenarios, and in time this may become an important part of the evaluation of pediatric patients with epilepsy. In some epilepsy syndromes, crucial interventions such as diet modification can have dramatic beneficial effects, so early diagnosis is vital [91, 92].

In our study, *SCN1A* was the main deleterious variant in Dravet syndrome and *KCNQ2*, *STXBP1*, *SCN2A* were found in Ohtahara syndrome. Deleterious variants in *STXBP1*, *KCNT1*, *CDKL5*, *ADSL* genes were found in West syndrome. Novel mutations in *SYNGAP1* were found in Doose syndrome, a *SLC2A1* mutation was found in GLUT1-DS and a *de novo* *MECP2* mutation were found in Rett syndrome. *TSC1/TSC2* variants were found in 60% of patients with tuberous sclerosis complex. Mutations found in unclassified epileptic encephalopathy were mainly in ion-channel genes. Thus, our study reinforces previous observations that the clinical syndrome and genetic etiology do not always match.

We tested 153 epilepsy genes and found 43 pathogenic and likely pathogenic variants in this study. Considering that over 500 epilepsy genes have been reported [73–76], our work was not comprehensive, which is a limitation of this study. With the decreasing cost of whole genome sequencing, the interrogation of the entire genome is now feasible for larger samples of epilepsy patients, and this approach has already been fruitful in other neuropsychiatric disorders such as autism, Kabuki syndrome, Bohring-Opitz syndrome and others [93, 94].

For genetic testing, it is proposed to conduct the strong candidate gene sequencing first (*SCN1A* for Dravet syndrome, *MECP2* for Rett syndrome and *TSC1/2* for tuberous sclerosis complex) before a NGS multi-gene panel testing [95–97]. In our study, we conducted targeted panel sequencing on Dravet syndrome and Rett syndrome patients before screening the strongest candidate gene for the following reasons. First, the correct clinical diagnosis of these syndromes can be difficult, especially in some of the younger patients in our sample, and often requires longitudinal assessment, which delays the correct diagnosis. Thus, we elected to perform NGS on our subjects before knowing the clinical diagnosis in some cases, such as these syndromes. Since our NGS panel that contains 153 epilepsy genes, our approach could facilitate the correct diagnosis in some cases. Second, it is now apparent that while 70–80% Dravet syndrome patients have *SCN1A* mutations, mutations in other genes such as *SCN1B, SCN2A, SCN8A, PCDH19, GABRA1, GABRG2, STXBP1, CHD*2 genes can cause Dravet syndrome like phenotypes [98], which would be missed if only *SCN1A* was sequenced. Similarly, *CDKL5* and *FOXG1* have been associated with atypical Rett syndrome [99], in addition to *MECP2*.

In tuberous sclerosis complex patients, we have a similar clinical scenario in which most features of tuberous sclerosis complex become evident only after 3 years of age, limiting their usefulness for early diagnosis [100]. In our study, all of the tuberous sclerosis complex patients’ initial presentations were seizures, of which 80% presented in the first year of life. 60% had hypomelanotic macules and 40% had multi nodules. 20% patient’s only clinical manifestation was seizures and 60% patients with seizures had only one major feature of tuberous sclerosis complex. 60% patients were found to have deleterious *TSC1* or *TSC2* mutations by NGS sequencing. Facial angiofibromas appeared by age 3–4 years in 60% patients in the follow-up period.

In summary, we identified 43 pathogenic or likely pathogenic variants, of which 26 mutations were novel and 32 were *de novo*. Variants in ion channel genes accounted for the largest category of gene in children with refractory epilepsy. Dravet syndrome is closely related to the *SCN1A* gene, which was the most frequently-appearing gene showing variants in our study. Novel and *de novo* mutations were found in Ohtahara syndrome, West syndrome, Doose syndrome and tuberous sclerosis complex pediatric patients. We also found a novel mutation in glucose transporter type 1 deficiency. Our results reinforce the importance and feasibility of precise genetic diagnosis for epilepsy, with the hope that in future, this will both aid in understanding the molecular pathophysiology and lead to new treatment targets.

## Additional file

Additional file 1: **Table S1.** The expression levels of the 153 targeted genes in brain. **Table S2.** The quality assurance (QA) /quality control (QC) of targeted sequencing. **Table S3.** The frequencies of different mutation locations in *SCN1A* gene and their corresponding phenotypes in Dravet syndrome patients. **Table S4.** The frequencies of different mutation types in *SCN1A* gene and their corresponding phenotypes in Dravet syndrome patients. (DOCX 98 kb)

## Abbreviations

ACMG: American College of Medical Genetics and Genomics
AEDs: antiepileptic drugs
EEOC: childhood-onset epileptic encephalopathy
EIEE: early-infantile epileptic encephalopathies
MT: Mutation Taster
NGS: next-generation sequencing
OMIM: Online Mendelian Inheritance in Man
PP2: PolyPhen-2
SCN1A: subunit alpha of sodium channel type 1
SIFT: Sorting Intolerant From Tolerant
