# Correction to: Novel and *de novo* mutations in pediatric refractory epilepsy

**DOI:** 10.1186/s13041-018-0399-y

**Published:** 2018-10-16

**Authors:** Jing Liu, Lili Tong, Shuangshuang Song, Yue Niu, Jun Li, Xiu Wu, Jie Zhang, Clement C Zai, Fang Luo, Jian Wu, Haiyin Li, Albert H C Wong, Ruopeng Sun, Fang Liu, Baomin Li

**Affiliations:** 1 Department of Pediatrics, Qilu Hospital of Shandong University, Jinan, Shandong People’s Republic of China; 2 Shandong University, Jinan, Shandong People’s Republic of China; 3 Qilu Children’s hospital of Shandong University, Jinan, Shandong People’s Republic of China; 4 MyGenostics Inc., Beijing, People’s Republic of China; 5 Campbell Family Mental Health Research Institute, Centre for Addiction and Mental Health, University of Toronto, Toronto, ON Canada

## Correction

Following publication of the original article [1], the authors reported that one of the authors’ names is spelled incorrectly. In this Correction the incorrect and correct author name are shown. The original publication of this article has been corrected.

Originally the author name was published as:

- Celement C. Zai

The correct author name is:

- Clement C. Zai
