# Supplementary material for: Novel and de novo mutations in pediatric refractory epilepsy
Source: Mol Brain. 2018 Sep 5;11:48. doi: 10.1186/s13041-018-0392-5 (PMC6125990; doi:10.1186/s13041-018-0392-5)
Supplement: Supplementary file 1 — Table S1. The expression levels of the 153 targeted genes in brain. Table S2. The quality assurance (QA) /quality control (QC) of targeted sequencing. Table S3. The frequencies of different mutation locations in SCN1A gene and their corresponding phenotypes in Dravet syndrome patients. Table S4. The frequencies of different mutation types in SCN1A gene and their corresponding phenotypes in Dravet syndrome patients. (DOCX 98 kb) [file 13041_2018_392_MOESM1_ESM.docx]

**Additional file 1**

**Table S1** **The expression levels of the 153 targeted genes in brain.**

| Category | | Number of genes |
| --- | --- | --- |
| Elevated expression in brain | Tissue enriched^*1^ | 21 |
|  | Group enriched^*2^ | 7 |
|  | Tissue enhanced^*3^ | 23 |
| Low expression in brain^*4^ |  | 14 |
| Medium expression in brain^*5^ |  | 88 |
| Total |  | 153 |

*1 At least five-fold higher mRNA levels in a particular tissue as compared to all other tissues.

*2 At least five-fold higher mRNA levels in a group of 2-7 tissues.

*3 At least five-fold higher mRNA levels in a particular tissue as compared to average levels in all tissues.

*4 The mean value of RPKM (Reads Per Kilobase Million) in brain <1.
*5 Genes that are not included in either elevated expression in brain group or low expression in brain group.

**Table S2 The quality assurance (QA) /quality control (QC) of targeted sequencing.**

| Case code | Raw_data_bases (Mb) | Clean_data_bases (Mb) | Aligned_bases (Mb) | Initial bases on target (bp) | Base covered on target (bp) | Effective bases on target (bp) | Average sequencing depth on target (X) | Fraction of target covered with at least 20X (%) | Duplication rate (%) |
| --- | --- | --- | --- | --- | --- | --- | --- | --- | --- |
| 1 | 581.01 | 574.83 | 573.54 | 560681 | 558061 | 211355911 | 376.96 | 94.63% | 21.74% |
| 2 | 582.59 | 574.09 | 572.37 | 560681 | 559325 | 154922545 | 276.31 | 98.03% | 19.56% |
| 3 | 538.05 | 534.15 | 533.38 | 560681 | 558297 | 192226698 | 342.85 | 90.79% | 25.79% |
| 4 | 653.64 | 649.02 | 647.77 | 560681 | 557904 | 216222303 | 385.64 | 91.48% | 27.14% |
| 5 | 575.87 | 568.79 | 567.47 | 560681 | 559628 | 184334613 | 328.77 | 95.22% | 14.14% |
| 6 | 581.16 | 575.54 | 573.42 | 560681 | 559094 | 189372341 | 337.75 | 95.20% | 16.88% |
| 7 | 482.49 | 476.6 | 475.47 | 560681 | 557489 | 162439201 | 289.72 | 90.73% | 21.48% |
| 8 | 838.78 | 827.59 | 825.35 | 560681 | 558915 | 273989434 | 488.67 | 96.31% | 27.28% |
| 9 | 802.01 | 793.62 | 792.36 | 560681 | 559722 | 331996711 | 592.13 | 95.76% | 16.92% |
| 10 | 461.89 | 426.51 | 425.74 | 560681 | 559457 | 136648618 | 243.72 | 95.87% | 18.82% |
| 11 | 1007.42 | 949.02 | 944.76 | 560681 | 560097 | 244781568 | 436.58 | 98.43% | 22.50% |
| 12 | 941.12 | 878.05 | 874.85 | 560681 | 559944 | 252670416 | 450.65 | 98.91% | 20.05% |
| 13 | 505.12 | 499.69 | 498.36 | 560681 | 558914 | 188176788 | 335.62 | 93.19% | 17.21% |
| 14 | 1089.46 | 1034.1 | 1030.69 | 560681 | 559923 | 286457371 | 510.91 | 98.36% | 18.11% |
| 15 | 799.01 | 769.98 | 768.43 | 560681 | 559587 | 294384423 | 525.05 | 93.76% | 18.20% |
| 16 | 578.96 | 571.68 | 569.47 | 560681 | 559153 | 192214818 | 342.82 | 93.63% | 23.72% |
| 17 | 327.81 | 312.39 | 310.67 | 560681 | 559084 | 131537875 | 234.6 | 97.29% | 15.34% |
| 18 | 1392.57 | 1280.01 | 1273.11 | 560681 | 560204 | 347420973 | 619.64 | 99.20% | 21.58% |
| 19 | 674.38 | 612.37 | 611.16 | 560681 | 559499 | 164097748 | 292.68 | 95.74% | 18.52% |
| 20 | 1795.39 | 1743.25 | 1741.56 | 560681 | 559925 | 700330064 | 1249.07 | 98.20% | 26.66% |
| 21 | 900.17 | 865.33 | 864.23 | 560681 | 560118 | 227598452 | 405.93 | 98.58% | 18.32% |
| 22 | 390.84 | 325.35 | 323.99 | 560681 | 559486 | 105014366 | 187.3 | 95.23% | 7.97% |
| 23 | 792.51 | 783 | 781.61 | 560681 | 558362 | 265595888 | 473.7 | 94.38% | 25.33% |
| 24 | 391.35 | 380.09 | 378.89 | 560681 | 559654 | 113979733 | 203.29 | 93.98% | 3.78% |
| 25 | 979.5 | 907.58 | 903.85 | 560681 | 560058 | 257515731 | 459.29 | 98.74% | 18.37% |
| 26 | 766.59 | 755.34 | 750.3 | 560681 | 558450 | 276325428 | 492.84 | 93.76% | 16.74% |
| 27 | 1074.89 | 995.53 | 991.59 | 560681 | 559856 | 272898289 | 486.73 | 99.06% | 20.40% |
| 28 | 611.4 | 565.86 | 561.9 | 560681 | 559938 | 144864223 | 258.37 | 98.47% | 20.82% |
| 29 | 695.81 | 666.43 | 665.68 | 560681 | 559859 | 208967792 | 372.7 | 98.55% | 15.88% |
| 30 | 671.22 | 662.44 | 658.23 | 560681 | 557594 | 259338211 | 462.54 | 91.50% | 18.90% |
| 31 | 958.09 | 921.15 | 920.19 | 560681 | 559782 | 238159075 | 424.77 | 98.69% | 18.25% |
| 32 | 704.46 | 673.12 | 672.44 | 560681 | 559981 | 209837132 | 374.25 | 98.49% | 16.58% |
| 33 | 516.07 | 483 | 480.1 | 560681 | 559639 | 140973202 | 251.43 | 98.45% | 18.80% |
| 34 | 638.27 | 632.35 | 630.59 | 560681 | 559243 | 216556024 | 386.24 | 95.32% | 22.64% |
| 35 | 544.46 | 538.46 | 537.3 | 560681 | 558933 | 190612016 | 339.97 | 93.16% | 21.44% |
| 36 | 835.53 | 797.23 | 795.83 | 560681 | 559779 | 183422967 | 327.14 | 98.47% | 18.70% |
| 37 | 548.57 | 528.93 | 527.3 | 560681 | 559343 | 147066342 | 262.3 | 98.21% | 6.93% |
| 38 | 578.06 | 570.7 | 569.43 | 560681 | 558258 | 190760337 | 340.23 | 91.40% | 28.30% |
| 39 | 1053.79 | 965.14 | 960.62 | 560681 | 559180 | 238379624 | 425.16 | 98.43% | 20.51% |
| 40 | 946.76 | 933.32 | 931.4 | 560681 | 559113 | 295066434 | 526.26 | 96.19% | 27.99% |
| 41 | 986.3 | 979.66 | 972.17 | 560681 | 558417 | 265714960 | 473.91 | 93.23% | 39.36% |
| 42 | 727.9 | 722.73 | 721.05 | 560681 | 559187 | 255840157 | 456.3 | 94.57% | 23.52% |
| 43 | 986.74 | 972.35 | 969.29 | 560681 | 559646 | 301209695 | 537.22 | 96.88% | 27.88% |
| 44 | 953.08 | 912.75 | 911.6 | 560681 | 559853 | 253309247 | 451.79 | 98.83% | 17.49% |
| 45 | 668.37 | 665.88 | 662.02 | 560681 | 559236 | 166380545 | 296.75 | 97.71% | 20.94% |
| 46 | 763.94 | 730.45 | 726.14 | 560681 | 559283 | 164051754 | 292.59 | 97.94% | 25.45% |
| 47 | 449.37 | 398.82 | 398.47 | 560681 | 559031 | 140435254 | 250.47 | 96.75% | 21.88% |
| 48 | 582.23 | 576.27 | 574.39 | 560681 | 558095 | 260822367 | 465.19 | 95.87% | 12.75% |
| 49 | 1151.36 | 1063.37 | 1059.37 | 560681 | 560101 | 204850075 | 365.36 | 98.37% | 16.18% |
| 50 | 884.22 | 879.49 | 876.89 | 560681 | 559310 | 212803361 | 379.54 | 98.33% | 18.78% |
| 51 | 1086.77 | 1083.19 | 1041.11 | 560681 | 559758 | 319051156 | 569.04 | 98.56% | 19.89% |
| 52 | 547.08 | 528.67 | 527.96 | 560681 | 559813 | 158614439 | 282.9 | 97.10% | 19.00% |
| 53 | 445.22 | 439.69 | 437.82 | 560681 | 558897 | 166219584 | 296.46 | 94.12% | 16.41% |
| 54 | 555.14 | 549.86 | 548.45 | 560681 | 559754 | 182583673 | 325.65 | 96.27% | 17.53% |
| 55 | 926.37 | 879.65 | 878.45 | 560681 | 559524 | 165947733 | 295.98 | 98.44% | 26.64% |
| 56 | 776.24 | 722.05 | 719.08 | 560681 | 558969 | 198456084 | 353.96 | 98.39% | 18.92% |
| 57 | 1439.99 | 1407.52 | 1405.11 | 560681 | 560458 | 530332923 | 945.87 | 99.16% | 29.43% |
| 58 | 1224.7 | 1220.15 | 1170.13 | 560681 | 559678 | 342337692 | 610.57 | 98.43% | 21.29% |
| 59 | 1810.91 | 1758.42 | 1752.75 | 560681 | 559630 | 168674071 | 300.84 | 95.99% | 15.72% |
| 60 | 530.88 | 523.44 | 521.84 | 560681 | 559047 | 209655635 | 373.93 | 95.29% | 23.87% |
| 61 | 709.35 | 658.65 | 654.39 | 560681 | 559894 | 179496436 | 320.14 | 98.81% | 20.46% |
| 62 | 1068.54 | 1064.13 | 1062.7 | 560681 | 560119 | 320516619 | 571.66 | 99.12% | 21.20% |
| 63 | 666.62 | 656.87 | 654.82 | 560681 | 559527 | 254538354 | 453.98 | 97.10% | 22.08% |
| 64 | 1483 | 1444.7 | 1442.56 | 560681 | 560191 | 596764503 | 1064.36 | 97.82% | 25.27% |
| 65 | 1097.08 | 1040.88 | 1037.91 | 560681 | 560358 | 259256636 | 462.4 | 99.11% | 18.66% |
| 66 | 572.17 | 566.46 | 565.35 | 560681 | 558636 | 203982827 | 363.81 | 94.64% | 21.39% |
| 67 | 578.35 | 574.06 | 572.5 | 560681 | 558500 | 207202677 | 369.56 | 94.26% | 23.57% |
| 68 | 367.47 | 351.35 | 350.01 | 560681 | 559373 | 124108827 | 221.35 | 97.58% | 19.37% |
| 69 | 361.34 | 343.6 | 343.2 | 560681 | 558984 | 92515830 | 165.01 | 94.90% | 19.05% |
| 70 | 1633.93 | 1577.03 | 1572.08 | 560681 | 559772 | 143392913 | 255.75 | 96.13% | 15.15% |
| 71 | 402.38 | 398.07 | 396.88 | 560681 | 558908 | 181159409 | 323.11 | 93.95% | 15.57% |
| 72 | 758.81 | 756.71 | 753.75 | 560681 | 559141 | 180991697 | 322.81 | 98.32% | 20.68% |
| 73 | 510.72 | 497.47 | 496.89 | 560681 | 558819 | 206389543 | 368.11 | 91.02% | 25.37% |
| 74 | 913.42 | 822.09 | 817.28 | 560681 | 559289 | 215650925 | 384.62 | 97.86% | 18.51% |
| 75 | 665.46 | 659.74 | 658.45 | 560681 | 559757 | 199650765 | 356.09 | 98.63% | 23.35% |
| 76 | 784.06 | 763.79 | 762.66 | 560681 | 559263 | 291615586 | 520.11 | 95.48% | 29.20% |
| 77 | 801.39 | 754.16 | 750.73 | 560681 | 559149 | 202908341 | 361.9 | 98.42% | 18.60% |
| 78 | 486.14 | 481.55 | 480.24 | 560681 | 558867 | 205779522 | 367.02 | 95.88% | 18.88% |
| 79 | 1171.18 | 1027.84 | 1023.29 | 560681 | 559918 | 296651013 | 529.09 | 98.26% | 21.62% |
| 80 | 609.78 | 586.69 | 584.47 | 560681 | 559134 | 169929229 | 303.08 | 96.89% | 19.64% |
| 81 | 675.82 | 667.69 | 666.27 | 560681 | 559449 | 248114745 | 442.52 | 96.10% | 20.42% |
| 82 | 655.68 | 653.52 | 631.41 | 560681 | 559276 | 146540642 | 261.36 | 97.59% | 13.23% |
| 83 | 1448.78 | 1443.58 | 1398.65 | 560681 | 559725 | 384051489 | 684.97 | 99.06% | 22.28% |
| 84 | 804.36 | 765.67 | 765.01 | 560681 | 560073 | 243486338 | 434.27 | 98.53% | 20.57% |
| 85 | 867.15 | 833.18 | 831.87 | 560681 | 559871 | 199703058 | 356.18 | 98.74% | 17.69% |
| 86 | 534.28 | 511.88 | 511.25 | 560681 | 559691 | 161567951 | 288.16 | 97.63% | 15.87% |
| 87 | 590.32 | 548.14 | 544.29 | 560681 | 559771 | 143259911 | 255.51 | 98.32% | 19.95% |
| 88 | 1064.31 | 1060.61 | 1014.17 | 560681 | 559800 | 302168447 | 538.93 | 98.42% | 20.23% |
| 89 | 1006.49 | 984.36 | 982.3 | 560681 | 560217 | 409857180 | 731 | 96.12% | 18.27% |
| 90 | 992.39 | 982.62 | 972.75 | 560681 | 560094 | 279638817 | 498.75 | 99.17% | 18.67% |
| 91 | 603.44 | 579.83 | 578.46 | 560681 | 559277 | 181150624 | 323.09 | 97.12% | 26.22% |
| 92 | 799.89 | 732.22 | 728.27 | 560681 | 559523 | 273291443 | 487.43 | 98.60% | 22.48% |
| 93 | 682.36 | 646.18 | 641.38 | 560681 | 559173 | 180392089 | 321.74 | 98.57% | 25.08% |
| 94 | 490.28 | 469.27 | 468.55 | 560681 | 559582 | 143507295 | 255.95 | 94.92% | 17.90% |
| 95 | 1498.93 | 1493.81 | 1450.16 | 560681 | 559714 | 407808379 | 727.34 | 99.10% | 21.77% |
| 96 | 1090.33 | 1078.78 | 1075.91 | 560681 | 560202 | 342282727 | 610.48 | 98.59% | 15.26% |
| 97 | 1470.08 | 1412.46 | 1405.49 | 560681 | 560255 | 463973749 | 827.52 | 99.66% | 29.90% |
| 98 | 828.86 | 824.5 | 823.15 | 560681 | 559094 | 274665574 | 489.88 | 98.78% | 23.32% |
| 99 | 813.37 | 806.56 | 804.68 | 560681 | 558444 | 286043514 | 510.17 | 94.64% | 25.18% |
| 100 | 955.62 | 919.39 | 915.53 | 560681 | 559544 | 315912864 | 563.44 | 99.13% | 21.27% |
| 101 | 908.66 | 854.5 | 850.75 | 560681 | 559681 | 304356402 | 542.83 | 98.86% | 21.75% |
| 102 | 807.1 | 750.71 | 748.5 | 560681 | 559532 | 225445479 | 402.09 | 98.78% | 12.12% |
| 103 | 544.09 | 523.87 | 521.38 | 560681 | 559680 | 185958178 | 331.66 | 98.38% | 20.84% |
| 104 | 519.31 | 491.73 | 486.92 | 560681 | 559237 | 153919443 | 274.52 | 97.93% | 17.75% |
| 105 | 735.57 | 698.86 | 697.96 | 560681 | 559787 | 260903573 | 465.33 | 98.92% | 17.09% |
| 106 | 560.87 | 536.92 | 534.53 | 560681 | 559452 | 181979109 | 324.57 | 98.48% | 19.41% |
| 107 | 715.81 | 683.07 | 680.35 | 560681 | 559694 | 225611992 | 402.39 | 98.76% | 20.51% |
| 108 | 641.25 | 613 | 610.63 | 560681 | 559329 | 213322366 | 380.47 | 98.67% | 19.55% |
| 109 | 1097.83 | 1033.44 | 1030.25 | 560681 | 559824 | 344722660 | 614.83 | 99.06% | 26.34% |
| 110 | 772.41 | 769.3 | 767.33 | 560681 | 559932 | 269332395 | 480.37 | 98.11% | 7.32% |
| 111 | 710.49 | 678.86 | 674.34 | 560681 | 559455 | 233747295 | 416.9 | 98.74% | 18.43% |
| 112 | 1100.45 | 1067.57 | 1061.04 | 560681 | 559895 | 343707386 | 613.02 | 99.11% | 18.28% |
| 113 | 733.37 | 676.16 | 673.78 | 560681 | 559380 | 198845854 | 354.65 | 98.61% | 17.99% |
| 114 | 681.84 | 641.12 | 638.64 | 560681 | 559611 | 206703336 | 368.66 | 98.10% | 20.94% |
| 115 | 839.08 | 789.31 | 787.4 | 560681 | 559918 | 206710566 | 368.68 | 98.54% | 21.62% |
| 116 | 375.76 | 351.07 | 349.94 | 560681 | 559032 | 135381734 | 241.46 | 96.72% | 20.66% |
| 117 | 754.23 | 716.38 | 714.29 | 560681 | 559817 | 214945331 | 383.36 | 98.32% | 20.03% |
| 118 | 489.51 | 466.45 | 464.61 | 560681 | 559369 | 167018047 | 297.88 | 98.21% | 22.25% |
| 119 | 378.97 | 360.12 | 358.76 | 560681 | 559239 | 129422109 | 230.83 | 97.27% | 21.27% |
| 120 | 588.07 | 566.93 | 564.51 | 560681 | 559821 | 181216089 | 323.21 | 98.71% | 20.93% |
| 121 | 557.3 | 447.16 | 444.43 | 560681 | 559477 | 157048237 | 280.1 | 97.50% | 18.94% |
| 122 | 700.39 | 528.14 | 527.52 | 560681 | 558896 | 172716012 | 308.05 | 97.74% | 35.86% |
| 123 | 886.42 | 828.81 | 827.78 | 560681 | 559366 | 229444238 | 409.22 | 98.50% | 26.74% |
| 124 | 924.07 | 899.11 | 897.65 | 560681 | 559368 | 316007287 | 563.61 | 93.79% | 34.56% |
| 125 | 1099.16 | 1018.12 | 1013.52 | 560681 | 560058 | 326183309 | 581.76 | 99.25% | 10.13% |
| 126 | 948.07 | 946.14 | 944.41 | 560681 | 559346 | 293731695 | 523.88 | 98.80% | 25.27% |
| 127 | 1189.36 | 1087.17 | 1082.55 | 560681 | 560194 | 354907516 | 632.99 | 99.34% | 10.25% |
| 128 | 320.3 | 301.2 | 300.69 | 560681 | 558988 | 119372036 | 212.91 | 96.93% | 16.04% |
| 129 | 732.39 | 649.33 | 647.85 | 560681 | 559371 | 238241223 | 424.91 | 98.30% | 17.53% |
| 130 | 744.23 | 709.53 | 708.17 | 560681 | 559511 | 270055739 | 481.66 | 98.08% | 14.24% |
| 131 | 683.67 | 638.63 | 637.68 | 560681 | 559754 | 255411640 | 455.54 | 98.60% | 21.53% |
| 132 | 779.49 | 735.26 | 733.72 | 560681 | 559905 | 169523740 | 302.35 | 97.04% | 18.74% |
| 133 | 763.52 | 710.22 | 708.6 | 560681 | 559752 | 260794930 | 465.14 | 98.84% | 19.17% |
| 134 | 364.04 | 344.7 | 344.34 | 560681 | 559087 | 91180921 | 162.63 | 93.88% | 20.01% |
| 135 | 1043.85 | 988.45 | 986.37 | 560681 | 560128 | 244609573 | 436.27 | 99.10% | 22.54% |
| 136 | 1232.77 | 1209.24 | 1206.3 | 560681 | 559887 | 443189012 | 790.45 | 97.71% | 29.77% |
| 137 | 529.16 | 514.92 | 514.09 | 560681 | 559608 | 155499146 | 277.34 | 97.43% | 18.56% |
| 138 | 771.3 | 744.33 | 743.09 | 560681 | 559726 | 277460273 | 494.86 | 94.02% | 20.11% |
| 139 | 517.06 | 511.86 | 510.54 | 560681 | 559651 | 228221050 | 407.04 | 95.71% | 11.42% |
| 140 | 494.41 | 447.32 | 446.85 | 560681 | 558799 | 175537685 | 313.08 | 97.05% | 24.56% |
| 141 | 633.43 | 577.13 | 572.62 | 560681 | 559233 | 134420231 | 239.74 | 98.03% | 11.88% |
| 142 | 562.77 | 534.48 | 533.14 | 560681 | 559529 | 125791772 | 224.36 | 96.71% | 17.55% |
| 143 | 691.63 | 657.42 | 655.97 | 560681 | 559613 | 251195858 | 448.02 | 97.31% | 14.36% |
| 144 | 592.53 | 590.8 | 589.22 | 560681 | 560067 | 227132396 | 405.1 | 98.29% | 7.27% |
| 145 | 687.86 | 685.96 | 684 | 560681 | 559971 | 257549896 | 459.35 | 98.32% | 8.20% |
| 146 | 563.59 | 562.73 | 560.85 | 560681 | 559707 | 229642217 | 409.58 | 98.86% | 9.95% |
| 147 | 648.42 | 612.23 | 611.37 | 560681 | 559631 | 241360868 | 430.48 | 98.69% | 21.48% |
| 148 | 1459.24 | 1327.24 | 1322.48 | 560681 | 560102 | 355037120 | 633.22 | 99.41% | 21.43% |
| 149 | 540.75 | 523.37 | 522.64 | 560681 | 559688 | 151345561 | 269.93 | 96.74% | 19.12% |
| 150 | 688.42 | 685.1 | 682.44 | 560681 | 559603 | 161474108 | 288 | 97.02% | 17.67% |
| 151 | 513.32 | 482.02 | 480.8 | 560681 | 559366 | 120435088 | 214.8 | 95.70% | 18.21% |
| 152 | 870.54 | 866.79 | 862.59 | 560681 | 560103 | 211621337 | 377.44 | 99.07% | 7.72% |
| 153 | 674.32 | 603.25 | 601.88 | 560681 | 558826 | 195244222 | 348.23 | 90.58% | 15.99% |
| 154 | 447.16 | 436.73 | 434.61 | 560681 | 559244 | 149572409 | 266.77 | 92.03% | 16.02% |
| 155 | 607.05 | 575.42 | 574.29 | 560681 | 559569 | 253984716 | 452.99 | 94.33% | 15.69% |
| 156 | 648.1 | 642.76 | 641.42 | 560681 | 559771 | 244191752 | 435.53 | 96.96% | 13.75% |
| 157 | 453.89 | 434.25 | 433.78 | 560681 | 560194 | 151399648 | 270.03 | 97.89% | 18.79% |
| 158 | 581.93 | 575.73 | 574.5 | 560681 | 556835 | 232296641 | 414.31 | 92.29% | 17.53% |
| 159 | 605.84 | 599.84 | 598.32 | 560681 | 559324 | 225459534 | 402.12 | 94.86% | 15.42% |
| 160 | 522.29 | 515.49 | 513.99 | 560681 | 559710 | 176133048 | 314.14 | 95.39% | 14.12% |
| 161 | 687.33 | 679.06 | 677.34 | 560681 | 559603 | 243161334 | 433.69 | 96.87% | 15.23% |
| 162 | 628.78 | 623.18 | 621.85 | 560681 | 559077 | 233812618 | 417.02 | 95.33% | 21.11% |
| 163 | 715.6 | 710.22 | 708.9 | 560681 | 558490 | 242333621 | 432.21 | 95.34% | 24.93% |
| 164 | 817.6 | 773 | 771.65 | 560681 | 559585 | 254487360 | 453.89 | 98.72% | 22.69% |
| 165 | 648.12 | 642.12 | 637.85 | 560681 | 557618 | 224529681 | 400.46 | 92.96% | 23.97% |
| 166 | 983.69 | 978.75 | 974.72 | 560681 | 559897 | 223021900 | 397.77 | 98.31% | 18.16% |
| 167 | 704.84 | 637.9 | 636.19 | 560681 | 558551 | 191609175 | 341.74 | 91.39% | 15.74% |
| 168 | 1381.91 | 1331.28 | 1326.15 | 560681 | 560213 | 499702243 | 891.24 | 98.03% | 19.04% |
| 169 | 903.84 | 892.57 | 890.6 | 560681 | 559005 | 278455256 | 496.64 | 95.97% | 28.68% |
| 170 | 855.31 | 683.89 | 677.9 | 560681 | 560146 | 175858505 | 313.65 | 98.21% | 8.82% |
| 171 | 809.13 | 804.3 | 802.76 | 560681 | 559811 | 198905498 | 354.76 | 98.39% | 21.58% |
| 172 | 1107.44 | 1075.28 | 1073.02 | 560681 | 559808 | 515188468 | 918.86 | 96.84% | 18.77% |

**Table S3** **The frequencies of different mutation locations in *SCN1A* gene and their corresponding** **phenotypes in Dravet syndrome patients.**

| Mutation Locations | | Total No. | Seizure types | | | | | | | | Family history | Abnormal Brain MRI | Developmental delay |
| --- | --- | --- | --- | --- | --- | --- | --- | --- | --- | --- | --- | --- | --- |
|  |  |  | FS | SE | Myo | FoS | GTCS | FBTC | aAb | C |  |  |  |
| In-frame | Intracellular | 1 | 1 | 0 | 1 | 1 | 0 | 1 | 0 | 0 | 0 | 0 | 1 |
|  | Extracellular | 3 | 3 | 2 | 3 | 2 | 2 | 0 | 1 | 0 | 1 | 0 | 3 |
|  | Transmembrane | 2 | 2 | 1 | 1 | 2 | 0 | 2 | 0 | 0 | 0 | 0 | 2 |
|  | Pore | 1 | 1 | 0 | 0 | 1 | 1 | 0 | 0 | 0 | 0 | 1 | 1 |
| Truncation* | Intracellular | 5 | 5 | 1 | 2 | 3 | 3 | 1 | 3 | 1 | 0 | 0 | 5 |
|  | Extracellular | 2 | 2 | 0 | 2 | 2 | 0 | 1 | 1 | 0 | 0 | 1 | 2 |
|  | Transmembrane | 1 | 1 | 0 | 1 | 1 | 1 | 0 | 1 | 0 | 0 | 0 | 1 |
| Splicing | Intracellular | 1 | 1 | 1 | 0 | 1 | 0 | 0 | 0 | 0 | 0 | 0 | 1 |

Abbreviations: FS, febrile seizures; SE, status epilepticus; FoS, focal seizures; FBTC, focal to bilateral tonic-clonic; Myo, myoclonic; aAb, atypical absence; GTCS, generalized tonic-clonic seizures; C, clonic; * Nonsense and frame-shift mutations are included in truncation mutations.

**Table S4 The frequencies of different mutation types in *SCN1A* gene and their corresponding phenotypes in Dravet syndrome patients.**

| Mutation Types | | Total No. | Seizure types | | | |  | | | | Family history | Abnormal Brain MRI | Developmental delay |
| --- | --- | --- | --- | --- | --- | --- | --- | --- | --- | --- | --- | --- | --- |
|  |  |  | FS | SE | Myo | FoS | GTCS | FBTC | aAb | C |  |  |  |
| Truncation | Nonsense | 4 | 4 | 0 | 2 | 3 | 2 | 1 | 2 | 1 | 0 | 0 | 4 |
|  | Frame shift | 4 | 4 | 1 | 3 | 3 | 2 | 1 | 3 | 0 | 0 | 1 | 4 |
| In-frame | Missense | 6 | 6 | 3 | 4 | 5 | 3 | 2 | 1 | 0 | 1 | 1 | 6 |
|  | Deletion | 1 | 1 | 0 | 1 | 1 | 0 | 1 | 0 | 0 | 0 | 0 | 1 |
| Splicing | | 1 | 1 | 1 | 0 | 1 | 0 | 0 | 0 | 0 | 0 | 0 | 1 |

Abbreviations: FS, febrile seizures; SE, status epilepticus; FoS, focal seizures; FBTC, focal to bilateral tonic-clonic; Myo, myoclonic; aAb, atypical absence; GTCS, generalized tonic-clonic seizures; C, clonic.
